# Autophagy-lysosomal pathway impairment and cathepsin dysregulation in Alzheimer’s disease

**DOI:** 10.3389/fmolb.2024.1490275

**Published:** 2024-10-31

**Authors:** Alquiandra Stefani Ferreira Mançano, Juliana Guanaes Pina, Bruna Rojas Froes, Juliana Mozer Sciani

**Affiliations:** Laboratório de Produtos Naturais, Universidade São Francisco, Bragança Paulista, São Paulo, Brazil

**Keywords:** Alzheimer’s disease, autophagy, lysosome, β-amyloid, cathepsin B, cathepsin D

## Abstract

Alzheimer’s disease (AD) is characterized by neuronal loss, attributed to amyloid-beta (Aβ) aggregation and accumulation. The autophagy-lysosomal pathway, including cathepsins B and D, is crucial for protein degradation and clearance, but it is impaired in some diseases. This review summarizes current knowledge on the dysregulation of this pathway in AD. Accumulating evidence suggests that Aβ overload impairs autophagy-lysosomal function and cathepsin activity, exacerbating Aβ accumulation and neurodegeneration. However, the precise mechanisms underlying these interactions remain elusive. Despite these challenges, targeting the lysosomal pathway emerges as a promising therapeutic strategy, and a comprehensive understanding of the autophagy-lysosomal system is essential to develop effective interventions for AD.

## 1 Introduction

The aging population has led to a significant increase in Alzheimer’s disease (AD) prevalence, making it a major public health concern, with projections estimating a doubling of cases every 5 years (D’Cruz and Banerjee, 2021). By 2050, this number is expected to soar to around 152 million (Janoutová et al., 2021).

AD is associated with the accumulation of amyloid-beta (Aβ) plaques and neurofibrillary tangles in the brain. Aβ peptides, mainly Aβ40 and Aβ42 fragments (Meng et al., 2024), are generated from the amyloid precursor protein (APP) through sequential cleavage by β- and γ-secretase, and are primarily cleared through the autophagy-lysosomal pathway (Breijyeh and Karaman, 2020). These peptides aggregate and accumulate to form plaques in various areas of brain tissue, and also contribute to the alteration of Tau protein, another cause of AD (Guan et al., 2021; Ge et al., 2022).

**FIGURE 1:**
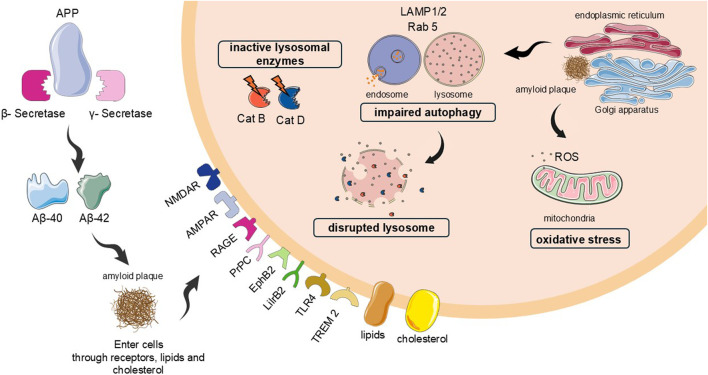
Implications of amyloid plaques in autophagy and lysosomal system, including cathepsins: the fusion of vesicles is impaired, reducing the autophagy, and the membrane of lysosome is disrupted, leaking the cathepsins D and B, the most abundant, to the cytosol, with inadequate pH for their better activity. These mechanisms contributed even more to the protein accumulation, and consequently neuron death. The disrupted lysosomes also cause release of reactive oxygen species (ROS), causing oxidative stress. Cat B and D = cathepsins B and D, respectively. ROS = reactive oxygen species.

These Aβ subunits are formed in intra and extracellular environment, and targeted to lysosomes and mitochondria (HAASS et al., 1993). Once Aβ is intracellular environment, it is internalized into autophagic vacuoles within lysosomes for subsequent degradation and elimination of these toxic compounds for the cells (Perez et al., 2014). Meanwhile, within mitochondria, the accumulation of these plaques on the inner membrane destabilizes it and disrupts the function of electron transport chain components, increasing reactive oxygen species (ROS) production (Milane et al., 2021; Song et al., 2023). However, it is observed that this autophagy-lysosomal system is not functional in AD, and amyloid plaques are not degraded, causing neuron’s death. Figure 1 depicted the action of amyloid peptides in autophagy-lysosomal system, including cathepsins and the consequences for mitochondria and neuron death.

The precise mechanisms underlying this lysosomal dysfunction are not fully understood, but it is believed to involve factors such as lysosomal membrane permeabilization related to ApoE4, impaired acidification, and reduced cathepsin activity, compromising this aspect of the proteostase system (Ji et al., 2002). The topic of lysosomal dysfunction in Alzheimer’s disease is controversial, as animal models may not fully recapitulate all aspects of the human disease, and cellular studies are often performed post-mortem. This review compiles these studies and results, as well as discussing medications that target autophagy currently in clinical trials for AD.

Due to its controversial nature, many studies investigating the role of the autophagy-lysosomal system in AD were discontinued or discouraged. However, recent studies increasingly highlight the importance of this topic in the disease and its potential as a key therapeutic target for novel treatments, including lysosomal cathepsins.

Given the critical role of the autophagy-lysosomal pathway in AD pathogenesis, a comprehensive understanding of this system is essential for developing effective therapeutic strategies. This review offers a comprehensive overview of AD, delving into its physiopathology, proteostasis, autophagy, lysosomal system, and the crucial function of cathepsins as lysosomal proteolytic enzymes. We examine how these systems contribute to the development and progression of AD.

## 2 Alzheimer’s disease - definition and physiopathology

Alzheimer’s disease (AD) was first described by Alois Alzheimer in 1906, who observed amyloid deposits and severe neuronal degeneration in the brain of a deceased patient. This pioneering work laid the groundwork for understanding the pathological hallmarks of the disease (Breijyeh and Karaman, 2020; Zhang et al., 2021).

AD is characterized by progressive neuronal loss in the hippocampus and cortex, leading to brain atrophy and cognitive decline. As the disease progresses, these changes become more pronounced, resulting in severe cognitive impairment. Neuropathological studies have identified a characteristic pattern of amyloid plaque accumulation, beginning in the pre-olfactory cortex and spreading to the entorhinal cortex, hippocampus, frontal, parietal, and temporal lobes (Tangaro et al., 2014; Nagata et al., 2016; Pini et al., 2016).

The onset of the patient’s symptoms is characterized by recent memory loss, and this condition worsens as the disease progresses. Over time, the patient begins to experience other symptoms such as behavioral and psychological changes, slurred speech, confusion, impaired judgment, and other abnormalities in brain function (Tarawneh and Holtzman, 2012).

The most accepted theory for the cause of AD is the synthesis and accumulation of Aβ. It is a peptide generated from the amyloid precursor protein (APP) through sequential cleavage by β- and γ-secretase enzymes. This process, termed the amyloidogenic pathway, produces primarily Aβ40 and Aβ42 variants. The acidic environment of endosomes and lysosomes is conducive to β-secretase activity. In contrast, the non-amyloidogenic pathway involves cleavage of APP by α-secretase, resulting in non-toxic fragments that inhibit Aβ formation and protect against AD (Meng et al., 2024).

β-amyloid fragments, especially the Aβ42 isoform, exhibit cytotoxic properties, particularly for neurons, also promoting the formation of ROS and induction of the release of inflammatory mediators in microglia (Forloni and Balducci, 2018; Jankovska et al., 2020).

Another component of AD is the Tau protein, responsible for promoting the specific assembly of the tubulin protein. In the pathogenesis of AD, the neurofibrillary tangles formed are the result of hyperphosphorylation of the Tau protein mediated by ROS, which can stimulate Tau protein hyperphosphorylation and disrupt calcium homeostasis in neuronal cell membranes, inducing the process of apoptosis (Guan et al., 2021; Ge et al., 2022).

While mitochondria possess intrinsic antioxidant systems, such as cytochrome c oxidase, their activity is significantly reduced in the AD hippocampus. This compromised antioxidant capacity renders mitochondria vulnerable to oxidative stress, leading to a vicious cycle of ROS generation and further mitochondrial damage. Such mitochondrial dysfunction is associated with the impairment of mechanisms that protect mitochondria against the action of ROS (Giorgi et al., 2018; Zhang et al., 2018).

Mitochondria possess antioxidant defenses, such as cytochrome c oxidase, and their activity is significantly reduced in the AD hippocampus (Gowda et al., 2022). Mitochondrial enzymes and complexes I, IV and V, besides pyruvate desydrogenase and α-ketoglutarate dehydrogenase complexes area altered in AD (Johri, 2021). This vulnerability, coupled with the generation of ROS, exacerbates mitochondrial damage, compromising the general metabolism and energy generation Furthermore, Aβ oligomers can directly infiltrate mitochondria via the ER-mitochondria interface or through the outer mitochondrial membrane (Huang and Liu, 2020). Their accumulation disrupts electron transport chain function, leading to a vicious cycle of increased ROS production and further mitochondrial impairment (Milane et al., 2021; Song et al., 2023).

The combined effects of Aβ accumulation and mitochondrial dysfunction contribute to impaired proteostasis and ultimately neuronal death.

## 3 Proteostasis

Proteostasis is the intricate process of maintaining protein homeostasis, and plays a pivotal role in safeguarding cellular health. This multifaceted mechanism encompasses three key systems: chaperones, the ubiquitin-proteasome system (UPS), and autophagy. These systems work in concert to ensure that proteins with faulty primary, secondary, or tertiary structures, as well as aggregated proteins, are either removed from the cell or properly refolded (Labbadia and Morimoto, 2015).

The endosome-lysosome system, a remarkable intracellular machinery, plays a pivotal role in maintaining cellular homeostasis by orchestrating the uptake of extracellular material and the intricate trafficking of macromolecules and organelles for recycling purposes. This intricate process, known as autophagy, serves as a cellular self-cleaning mechanism, ensuring the proper functioning and survival of cells (Vilchez et al., 2014).

### 3.1 Autophagy

Autophagy is a cellular degradation process essential for proteostasis, and it is initiated by nutrient deprivation, cellular stress or the presence of misfolding/mutated/aggregated proteins. There are four primary types:

(1) Macroautophagy involves the formation of double-membrane structures called autophagosomes, which engulf cellular components and fuse with lysosomes for degradation (Feng et al., 2014).
(2) Microautophagy is a more selective process where cellular components are directly engulfed by lysosomes. This can occur through various mechanisms, including chaperone-mediated autophagy and peroxisome degradation (peroxophagy) (Kunz et al., 2004).
(3) Chaperone-mediated autophagy, for degradation of cytosolic proteins, targeting them to the lysosomes (Kaushik and Cuervo, 2012).
(4) Mitophagy is a specialized form of autophagy targeting damaged mitochondria, facilitating their clearance and preventing cellular dysfunction (Gómez-Sintes et al., 2016).

Thus, lysosomes, the central components of this degradation machinery, are acidic organelles (pH 4.5–5.5) containing hydrolytic enzymes, primarily cathepsins (Saftig and Klumperman, 2009). They fuse with endosomes and autophagosomes, delivering their contents for degradation. This intricate network, encompassing endocytosis, autophagy, and lysosomal activity, is vital for recycling cellular components, generating energy, and maintaining cellular health (Yorimitsu and Klionsky, 2005).

Endocytosis brings extracellular substances into the cell within vesicles that mature into early and late endosomes before fusing with lysosomes. Autophagy delivers intracellular components for degradation, and the intricate interplay between these pathways, often referred to as the “lysosomal network,” is essential for cellular homeostasis. This network not only recycles cellular components and generates energy but also provides critical information about the cell’s internal and external environment (Nixon, 2013).

Macromolecules or ligands enter in the cell through present in clathrin regions, composed by plasma membrane, to then be delivered to the lysosomes. This process regulates the interaction of cells and provides energy by capturing substrates important for molecule synthesis (Doherty and McMahon, 2009).

The autophagy pathways rely on important proteins for vesicle fusion and transport. Rab5a regulates the fusion of endocytic vesicles with early endosomes. Its function, as proposed by de Hoop et al. (1994), is primarily related to the regulation and fusion of early endocytosis, playing a crucial role in the formation and transport of endosomes, cellular structures responsible for the internalization of cell membrane and surface proteins. Additionally, Rab5a may also be involved in the recycling of membrane components from synaptic vesicles in neurons, through its association with early endosomes both in dendrites and axons (de Hoop et al., 1994).

Another important protein for autophagy is LAMP, a lysosome-associated membrane protein. These membrane proteins of types 1 and 2 (LAMP-1 and LAMP-2) associate and stabilize polypeptide translocation systems, such as the transporter associated with antigen processing (TAPL). Dysfunction in the autophagy-lysosomal pathway of neurons, as seen in diseases like Alzheimer’s, leads to exosomal uptake of lysosomal membrane proteins and lysosomal granule enzyme cathepsin D. Consequently, levels of LAMP-1 are elevated in neural-derived plasma exosomes from patients with AD compared to those without the disease, as will be commented below (Goetzl et al., 2015). Moreover, LAMP-2A also is a receptor for intracellular protein transport in the chaperone-mediated autophagy (Qiao et al., 2023).

Lysosomes contain several hydrolases for a wide range of substrates, such as DNA, RNA, proteins, and lipids. However, the main ones being cathepsins. Its environment is acidic due to the action of the proton pump vacuolar-type ATPase, localized into the lumen of lysosomes, which ensuring the maintenance of pH, and consequently the proper functioning of the organelle (Turk et al., 2012; Futai et al., 2019). In mice, G subunit isoforms (G1 and G2) were found in neurons, strongly expressed in cortical and hippocampal areas, important for the acidification of organelles and synaptic vesicles (Murata et al., 2002). In general, neurons are rich in lysosomes, as this cell type needs to remove proteins through autophagy, maintaining proteostasis. Studies show that the reduced autophagy is related to neurodegeneration (Song et al., 2020).

The correct acidification of lysosomes and good functioning of the organelle reflects on mitochondrial function. The control of mitochondrial processes is essential for protecting the cell from aging and related diseases, such as dementia. Abnormal mitochondria are removed by autophagy and mitophagy defects are linked to mitochondrial dysfunction (Mindell, 2012).

## 4 Cathepsins

Cathepsins are peptidases belonging to the families of the serine peptidases (cathepsins A and G), aspartic peptidases (cathepsins D and E) or cysteine peptidases (cathepsins B, C, L, F, H, K, O, S, V, X and W), which are different in their structures, cellular and tissue localization and expression (Saftig et al., 1998). They are related to several biological processes, including lysosomal activities (Brömme, 2000).

Cathepsins are initially inactive and require activation through proteolytic removal of the N-terminal propeptide (Neurath, 1999). This activation process is crucial for triggering their biological functions, allowing them to play important roles in various physiological and pathological pathways, including protein digestion, antigen processing, and tissue remodeling (Sonkar et al., 2022).

Once activated, they play crucial roles in protein turnover. Beyond their degradative functions, cathepsins are involved in diverse cellular processes, such as angiogenesis and apoptosis (Premzl et al., 2006). Cathepsin B, for instance, participates in mammary gland involution and is implicated in lysosome-mediated cell death (Kreuzaler et al., 2011). Overexpressed cathepsins B and L, regulated by the transcription factor Stat3, can be released into the cytosol, where they act as potent proteases, triggering apoptosis (Lockshin and Zakeri, 2004). While distinct from classical apoptosis, this process involves the activation of pro-apoptotic proteins such as caspases (Boya and Kroemer, 2008).

The multifaceted functions of cathepsins highlight their significance in both physiological and pathological processes, underscoring their potential as therapeutic targets for various diseases (Cunha et al., 2022).

Interestingly, cathepsin D also influences cellular processes beyond its proteolytic function. Berchem and colleagues reported its mitogenic and angiogenic effects in cancer cells. While the exact mechanisms remain unclear, both catalytically active and inactive forms of cathepsin D contribute to tumor growth and progression (Berchem et al., 2002).

## 5 The endosomal-lysosomal pathway and AD

Neurons heavily rely on this autophagy system, given that the brain is often the organ most severely affected in primary lysosomal disorders. Consequently, mutations in genes involved in the global lysosomal network are causally linked to neurodegenerative disorders frequently (Komatsu et al., 2006).

Neurons possess extensive dendritic and axonal cytoplasmic extensions, which is why they face specific challenges in preventing dysfunctional organelles and cellular debris that may accumulate throughout life without the assistance of cellular division. Young neurons efficiently perform this task by eliminating autophagic substrates (Boland et al., 2008). However, the long distances that the many autophagic vacuoles generated in axons must travel to reach lysosomes, which are primarily concentrated near the cell body, it is not surprising that neurons are particularly vulnerable to slowdowns in the proteolytic clearance of autolysosomal substrates (Lee et al., 2011). In the absence of competent autophagy, neurons accumulate protein aggregates and degenerate (Hara et al., 2006; Komatsu et al., 2006). In neurodegenerative diseases, autophagy goes awry at various points along the way, giving rise to distinct pathologies (Nixon, 2013).

Therefore, lysosomal alterations and LCD (lysosomal cell death) have been identified as key pathological events in diseases that develop with age and neurodegenerative diseases (Serrano-Puebla and Boya, 2016). In AD, several alterations in proteostasis, the ubiquitin-proteasome system, autophagy, and chaperones have been observed. One of the compartments where Aβ is generated through sequential cleavages of APP is the endoplasmic reticulum (ER), in addition to the medial and trans-Golgi complex, where it is compartmentalized and exported to other cellular locations. Aβ40 is exclusively generated in the trans-Golgi portion, while Aβ42 is generated within the ER before reaching the Golgi via secretory vesicles. The amyloid peptide, whether generated intra- or extracellularly, is recognized as abnormal, and in the cytosol, it undergoes ubiquitination to be targeted either to the proteasome for degradation, to macroautophagy or to chaperone-mediated autophagy (CMA) (Choy et al., 2012; Mputhia et al., 2019).

Extracellular Aβ oligomers can enter in the cells by interacting to lipids and cholesterol from neuron’s membrane. However, it has been well described that the monomers, oligomers or fibrils bind to cellular receptors, such as NMDAR and AMPAR (Zhang et al., 2023). Other receptors also are important for Aβ binding, such as RAGE receptor (receptor for advanced glycosylation end-products), responsible for capturing circulating Aβ to the brain (Deane et al., 2003; Jarosz-Griffiths et al., 2016).

Cellular prion protein (PrPC) and Ephrin receptor B2 (EphB2) have been identified as Aβ receptors, and downstream signaling of both alters N-methyl-D-aspartate (NMDA) receptor function (Laurén et al., 2009; Cissé et al., 2011; Um et al., 2012) Furthermore, LilrB2, a neuron surface receptor, associated with growth cones and synapses, has been shown to capture oligomeric forms of Aβ42 (Cao et al., 2018). Toll-like receptor 4 (TLR4) are the main receptor in microglia that interacts to aggregated Aβ to activate the cell, activating the complement system, NLRP3 inflammasome and release of cytokines (Yang et al., 2020).

Another receptor, called TREM2, is a single-pass transmembrane structure expressed in microglia and myeloid cells 2, binds to a variety of ligands, for example, lipoproteins, phospholipids and oligomeric Aβ (oAβ) (Wang et al., 2022). A study conducted by Zhao et al. (2018) showed that TREM2 binds directly to β-amyloid (Aβ) oligomers with nanomolar affinity, and its deficiency impairs Aβ degradation in primary microglial culture and in the mouse brain.

Marshall et al. (2020) showed that oligomeric Aβ binds to extracellular membrane and also is internalized in rats’ primary neurons through plasma membrane to reach cytoplasm, in a time-dependent manner. Moreover, the oligomeric peptide is endocyted by dynamin-dependent mechanism, being addressed to lysosomes. Interestingly, Aβ42, when reach lysosomes, impair its function, influencing the traffic of other proteins inside the cell.

The uptake of oligomeric Aβ was observed using 10 μM of the peptide in the Marshall’s study, but was also demonstrated with 1 μM in SH-SY5Y neurons. Hu et al. (2009) showed that 1 nM of extracellular Aβ could be uptaken and accumulated in lysosomes in concentration higher than 2.5 μM, enough to impair the organelle activity.

The ubiquitin-proteasome system activity is compromised in aging, a common condition in AD patients, and therefore not a feasible pathway for Aβ removal. The main consequences of Aβ and Tau oligomer presence in the cells that impair the proteasome activity is its localization and accumulation of polyubiquitinylated substrates (Tai et al., 2012). Continuous proteasome inhibition leads to excessive activation of the lysosomal system, resulting in increased autophagy (Ihara et al., 2012). Thus, intracellular Aβ is internalized into autophagic vacuoles containing apolipoprotein E (ApoE) for subsequent degradation, while misfolded Tau protein is delivered to the same organelle by chaperone-mediated autophagy (Fuentealba et al., 2010; Caballero et al., 2018). Many studies describe the secretory pathway from the endoplasmic reticulum (ER), Golgi, and endosomal pathway in APP processing for Aβ generation and transport, including the processing of both Aβ40 and Aβ42. Early endosomes are identified as the primary targeting site for Aβ (Perez et al., 2014).

Lysosomes, in turn, are responsible for intracellular degradation of this Aβ, ensuring cellular homeostasis maintenance, as previously discussed. However, in the context of AD, there is dysfunction in autophagy, which can lead to even greater accumulation of Aβ in neurons, further contributing to neuronal death (Reddy and Oliver, 2019).

There is substantial evidence indicating that one mechanism for autophagy disfunction in AD is the lysosomal rupture. One piece of evidence is related to ApoE4, which is the strongest genetic risk factor for late-onset AD and functions as a lipid transport protein. ApoE4 has been shown to destabilize lysosomal membranes. It is proposed that in the presence of amyloid peptide, ApoE4 forms a reactive molecular intermediate that binds to phospholipids and integrates into the lysosomal membrane. This integration destabilizes the membrane, leading to lysosomal leakage and ultimately apoptosis (Perez et al., 2014).

Besides, when the Aβ is present in early endosomes, they become enlarged, reinforcing the importance of the endocytic pathway in AD. This pathway is related to the internalization of both Aβ and ApoE (Vidoni et al., 2016). Moreover, the accumulation of Aβ42 in the lysosomes of AD neurons leads to lysosomal leakage and the release of enzymes into the cytoplasm, both of which correlate with morphological evidence of cellular toxicity (Yang et al., 1998).

In aging, senescence or dementia, there is dysfunction of the v-ATPase or disruption of the lysosome membrane, leading to alkalinization of the organelle, compromising the catalytic activity of the enzymes present. In the case of lysosome disruption, the extravasated protons decrease the pH of the cytosol, altering cellular metabolism. Without hydrolysis, there is no elimination of undesirable proteins and lipofuscin stores, and consequently the accumulation of metabolites and cellular debris. Some compounds and elements, such as the amino acid cysteine, reduce the availability of iron ions, compromising mitochondrial respiration, besides the lysosome (Mindell, 2012).

Early onset AD can be caused by presenilin 1 mutations, which alter lysosomal proteolysis and autophagy (Lee et al., 2010) leading to calcium dyshomeostasis due to impaired V-ATPase-mediated lysosomal acidification (Lee et al., 2015). Increases in calcium levels, calpain activation, and LCD are observed in many models of neuronal cell death including excitotoxicity and stroke (Yamashima and Oikawa, 2009).

Kim et al. (2023) showed a destruction of endolysosomes by neurons rich in Aβ and brains from AD patients, related to the impairment of the V-ATPase activity. Interestingly, a gene suppressor reverted the lysosomal dysfunction and this intervention could increase the memory of transgenic mice for AD.

In addition to this mechanism of lysosomal disruption, there are studies showing alterations in autophagy and proteostasis pathways in AD. Decreased levels of Hsp70 and increased levels of cathepsin D, LAMP-1, and ubiquitinated proteins are found in neurally-derived exosomes taken from AD patients prior to the onset of clinical signs (Goetzl et al., 2015). Furthermore, alterations in mTOR signaling and reduced autophagy in postmortem tissue from AD patients correlate with decreased expression of the autophagy regulator Beclin1 (Tramutola et al., 2015). Collectively, these observations indicate alterations occurring at multiple levels within the endolysosomal system in AD patients.

The alteration in proteins from autophagy pathway was also observed in a study that analyzed the brains of pre-AD patients, who had the presence of Aβ but no clinical manifestations of the disease, compared to age-matched controls without the comorbidity or presence of the peptide. In control patients, endosomes appeared normal in size, as visualized by an early endosome marker, anti-rab5. In pre-AD patients, however, endosomes in hippocampal and prefrontal cortex neurons were enlarged. Another marker, rabaptin 5, which regulates endosomal anchoring and fusion, was observed in the cytosol of control cells but was found within endosomes in the pre-AD group. Additionally, an increase in Rab4, a protein involved in directing the recycling of early endosomes to the cell surface, was observed in these patients (Tramutola et al., 2015). These endosomal abnormalities were more pronounced in patients with the ε4 allele and even more so in patients with late-onset FAD-APOE ε4 and Down Syndrome, who develop AD in aging due to Aβ production, as well as in patients with PS mutations causing early-onset (Cataldo et al., 1997).

The results from analyses of plasma-derived neuronal exosomal proteins, derived from lysosomes and related cellular organelles, enhance the understanding that lysosomal dysfunction in AD may provide useful biomarkers for the recognition of preclinical AD (Goetzl et al., 2015).

## 6 Cathepsins and Alzheimer’s

Cathepsins have been demonstrated to have a strong relation in the physiopathology of diseases, including Alzheimer’s (Jung et al., 1999; Lynch and Bi, 2003).

Levels of cathepsin D are increased in AD and we found in extra lysosomal localization in brains from old rats (Jung et al., 1999; Lynch and Bi, 2003). Also, an increase in cathepsin B and E activities have been found with age, whereas a decrease in cathepsin L is observed (Nakanishi et al., 1994). Moreover, these changes in cathepsin levels and localization are common to neurons and glial cells in the brain and are restricted to areas in the brain with increased vulnerability to age related diseases (Nakanishi et al., 1994; Jung et al., 1999; Lynch and Bi, 2003).

Interestingly, extracellular accumulations of cathepsins B, L, and D associated with Aβ deposits have been described in the brains of patients with AD, but not in other neurodegenerative diseases such as Parkinson’s or Huntington’s disease. Specific sections from AD patients display cathepsin antigens intracellularly within lysosomes and related structures, as well as extracellularly in senile plaques, within degenerated neurons (non-neuronal cells with plaques do not show labeling). Cathepsins B and D are associated with amyloid deposits within extracellular lipofuscins. Controlled patients (without the disease) showed no reactivity (Cataldo and Nixon, 1990).

Cathepsins are known to activate and/or degrade various important neuronal proteins, thus playing significant roles in neurodegenerative disorders (Stoka et al., 2016; Vidoni et al., 2016).

### 6.1 Cathepsin D

Lysosomal cathepsin D is a fundamental aspartic endopeptidase for cellular processes, meticulously regulated by a series of intracellular mechanisms. These include enzyme synthesis regulation, post-translational modifications, activation of the pro-enzyme form, and maintenance of lysosomal pH. The enzyme is synthesized in an inactive form, and a further processing for an N-terminal propeptide removal results in its active form and an enzyme of 48 kDa (Benes et al., 2008).

Cathepsin D plays a crucial role in neuronal homeostasis, and its dysfunction leads to impaired proteolysis of target proteins such as huntingtin, α-synuclein, Tau, lipofuscin, apoE, which can result in neurological diseases including Parkinson’s and Alzheimer’s, among others (Vidoni et al., 2016).

In general, proteolysis mediated by cathepsin D is essential for neuronal cell homeostasis through the degradation of unfolded or oxidized protein aggregates delivered to lysosomes via autophagy or endocytosis. Specifically, many altered neuronal proteins characteristic of neurodegenerative diseases (such as APP, α-synuclein, huntingtin) are physiological substrates of cathepsin D and would accumulate abnormally if not degraded by this enzyme. Furthermore, experimental evidence indicates that cathepsin D activity is linked to cholesterol and glycosaminoglycan metabolism, which explains its involvement in plasticity (Vidoni et al., 2016).

Nakamura et al. (1991) reported an abnormal distribution of both cathepsin D and cathepsin B in brains of early-onset AD patients, indicating that the lysosomal proteases could be altered in the disease.

Cataldo et al. (1994) showed that the lysosomal system is altered in AD patients, being the disturbance related to the progression of the disease. In this study, authors showed that the cathepsin D immunoreactivity was increased according to the quantity of Aβ.

Lysosomal cathepsin D plays a crucial role in degrading unfolded protein aggregates delivered through endocytosis or autophagy, relevant for diseases that are caused by protein accumulation, such as AD (Vidoni et al., 2016).

Besides this overlapping between amyloid plaque and cathepsin D, and the similarity to peptidases that produce Aβ, an important question emerged: has the enzyme a role in the APP processing, and consequently formation of Aβ or did the enzyme could process and eliminate the β-amyloid peptide and amyloid plaque?

The APP from the endolysosomal system is cleaved by acidic peptidases, generating Aβ, secreted to the extracellular media, and also addressed to lysosomes. Such enzymes are β and γ-secretases, with share common features of cathepsin D (Haass et al., 1993).

Schechter and Ziv (2008) demonstrated that the kinetic parameters of cathepsin D and BACE-1 are similar, using the APP as substrate (β-sites of the wild type or mutated Swedish families), but both with slow cleavage, with (kcat/Km: approx. 50 m^−1^ s^−1^). On the other hand, in the human brain, cathepsin D was 280-fold more abundant compared to BACE-1 and was inhibited by pepstatin A, in contrast to BACE-1, which was not inhibited, showing important differences between them.

Higaki et al. (1996) demonstrated that cathepsin D and γ-secretase do not have the same cleavage site of two recombinants APPs (containing 156 and 100 amino acids of its carboxyl terminus). Interestingly, they showed that the cleavage of APP by the cathepsin D changes according to the tridimensional structure of the substrate. Processing of beta-amyloid precursor protein by cathepsin D was demonstrated by Higaki.

Previous studies had shown similar results Dreyer et al. (1994), Gallwitz et al. (2022) showed that cathepsin D could cleave APP, but under some parameters: the enzyme hydrolyzed APP between Glu593 and Val594 residues. Interestingly, they showed that the velocity of the reaction increased according to the mutations of APP, such as Asn instead Lys595 and Leu instead Met.

Therefore, cathepsin D can generate Aβ species in some circumstances, but not in the amyloidogenic pathway, being not relevant for the disease cause or development. Moreover, considering that there are reported several variants for APP, cathepsins and secretases, further studies have to be performed to confirm the activity of cathepsin D as a APP processing (Schechter and Ziv, 2008).

On the other hand, it is well described that the cathepsin D is able to cleave Aβ40 and 42. Gallwitz et al. (2022) demonstrated that cathepsin D cleaved Aβ42, without affecting the intracellular APP processing and consequently Aβ formation by *in vitro* and *in vivo* models.

This data has been earlier demonstrated by McDermott and Gibson (1996), Hamazaki (1996) and by Kenessey et al. (1997), which showed the ability of cathepsin D in the processing of Tau protein as well, using *in vitro* technique. Moreover, the deletion of the cathepsin D gene caused an increase (x4) of intracerebral Aβ Suire et al. (2020) confirming the correlation between the enzyme activity and the amyloid peptide processing.

Studies show the upregulation of cathepsin D in the brain from AD patients. Elevated levels of cathepsin D have also been observed in amyloid plaques in the brain and cerebrospinal fluid (CSF) of AD patients. However, studies on cathepsin D levels in the plasma of AD patients have shown inconsistent results. While studies show increased levels of cathepsin D in plasma samples, others report negative regulation in fibroblasts and monocytes derived from AD patients (Kim et al., 2021).

In the study conducted by Kim et al. (2021), using immunoblotting and ELISA techniques, a reduction in plasma levels of cathepsin D was observed in patients with amyloid defects (AD group). Furthermore, a negative correlation was found between plasma levels of cathepsin D and the CDR-SB score, one of the clinical cognitive standards commonly used in AD diagnosis. On the other hand, cathepsin D was found in high levels in plasma from AD patients, presenting cognitive decline and brain atrophy. These findings suggest that plasma levels of cathepsin D may serve as a novel biomarker for the diagnosis of AD (Drobny et al., 2022; Chai et al., 2023).

This overexpression of cathepsin D is probably due to the impaired activity of the enzyme. Cathepsin D was found oxidized in the AD brain, compared to healthy people. Moreover, the oxidized V-ATPase was also found, which indicates that the impairment of cathepsin D enzymatic activity is related to the incorrect lysosome acidification (Di Domenico et al., 2013). This is an important aspect, considering that cathepsin D is the primary soluble protease that degrades Aβ at acidic pH (Benes et al., 2008).

Loss-of-function mutations and variations of cathepsin D lead to multiple forms of neurodegeneration in humans (Steinfeld et al., 2006) and is related to the risk of late-onset AD (Davidson et al., 2006).

Suire et al. (2020) demonstrated a critical role for cathepsin D in maintaining cerebral proteostasis. CatD-knockout (CatD-KO) mice exhibited increased levels of insoluble Aβ42 and Aβ40, correlating with a reduction in soluble Aβ species. This suggests that cathepsin D is the primary protease responsible for degrading soluble Aβ within the brain. Notably, the absence of cathepsin D did not affect the levels of APP, its C-terminal fragments, or other major Aβ-degrading enzymes like IDE and NEP, indicating a specific role for de enzyme in Aβ clearance. The accumulation of insoluble Aβ within lysosomes of CatD-KO mice further supports the hypothesis that CatD is crucial for Aβ degradation in this organelle. Interestingly, the inverse relationship between soluble and insoluble Aβ levels suggests that Aβ42 may inhibit cathepsin D activity.

Thus, cathepsin D has an important function of eliminating Aβ42, as a protective (Suire et al., 2020) rule in the AD, but the enzyme activity may be compromised, increasing the risk of the disease.

A large number of genetic association studies have investigated a non-synonymous single nucleotide polymorphism (SNP) located in exon 2 (C to T substitution leading to the amino acid change from alanine to valine) of the cathepsin D gene (CTSD), which has previously been associated with AD, where the T allele increases the risk of developing AD. This finding is consistent with previous *in vitro* studies that linked the CTSD^*^T allele to increased pro-cathepsin D secretion and alteration of the enzyme’s intracellular maturation (Touitou et al., 1994).

The association of the CTSD polymorphism with AD was replicated in a subsequent study involving the Irish population (Touitou et al., 1994).

Crawford et al. (2000) reported an ethnicity-dependent association of the CTSD polymorphism with AD, where the CTSD∗C allele was significantly overrepresented in Hispanic patients with AD.

Menzer et al. (2001) did not detect a significant association in the overall population of German patients with AD and control individuals. However, they reported significant results in interactions between APOE4 and CTSD∗T alleles in men. Additionally, they found that the CTSD∗T allele exerts a significant influence on dementia-free survival.

However, two studies in the United States failed to confirm the association between the DA genotype and CTSD (Bhojak et al., 2000; Bertram and Tanzi, 2001). In order to clarify these findings, Papassotiropoulos et al. (2002) measured the concentration of Aβ42 and Aβ40 peptides in patients and control individuals. As a result, the CTSD genotype T allele was associated with a 50% reduction in Aβ42 levels in cerebrospinal fluid.

Discrepancies among genetic association studies are common and can be attributed to various causes: initial findings may be false positives due to multiple testing or population heterogeneity, negative studies may result from low statistical power or population admixture, or initial findings may be true only in certain populations (Suire et al., 2020). As stated by Roses, (1998) genetic associations should be validated by assessing their biological relevance to the disease.

Thus, when considered individually, these studies yielded conflicting results. However, utilizing data from AlzGene Bertram et al. (2007) a meta-analysis of all 18 reports exclusively from Caucasian populations published to date, excluding those with Hardy-Weinberg and balance violations, yielded a statistically significant result (Suire et al., 2020).

### 6.2 Cathepsin B

Cathepsin B is a cysteine protease enzyme able to degrade peptides and proteins that achieve the lysosomal pathway by endocytosis or phagocytosis (Chapman et al., 1997), playing a crucial role in the degradation of intracellular proteins. Its presence is widely observed in a variety of human cells (Cunha et al., 2022) and is primarily located in the lysosomal and endosomal compartments (Sun et al., 2008). However, it has the ability to act both intracellularly and extracellularly, in various types of cells. The enzyme plays an important role in AD, as it can degrade oligomeric Aβ42 and is subsequently secreted by exocytosis (Mueller-Steiner et al., 2006). Thus, it could help to remove Aβ from the extracellular environment or attenuate the intracellular toxicity of Aβ, which has been associated with the accumulation of Aβ1-42 aggregates in endosomal vesicles and other cellular organelles (Zhang et al., 2002; Esposito et al., 2004; Takahashi et al., 2004; Oddo et al., 2006).

Therefore, cathepsin B emerges as a promising target for therapeutic interventions in AD. Substantial research implicates significant involvement of category B in the cognitive and behavioral deficits associated with AD. They also highlight a comprehensive approach to clinical studies and animal models of AD, including the use of cathepsin B gene knockout and chemical inhibitors, highlighting the importance of this enzyme for new therapeutic approaches that could be used in AD (Hook et al., 2020).

Cathepsin B possesses the unique ability to act both as a dipeptidyl carboxypeptidase and as an endopeptidase. This is due to having two histidine residues (His110 and His111) in a 20-residue occlusion loop on the primer side of its catalytic site (Khouri et al., 1991; Illy et al., 1997; Nägler et al., 1997). This segment called the “occlusion loop,” plays a crucial role in regulating enzymatic activity, triggered as an autoinhibition mechanism. When not involved in proteolytic manipulation processes, the occlusion loop blocks the active site of cathepsin B, preventing interaction with its substrates. This fine-tuned adjustment helps prevent indiscriminate manipulation of cellular proteins and contributes to protein homeostasis within the cell (Cunha et al., 2022).

Furthermore, optimal activity of cathepsin B occurs in an acidic environment, with pH between 4.5 and 5.0, consistent with its primary activity site in lysosomes, where pH is low (Cunha et al., 2022).

However, cathepsin B is also found in the extracellular environment (Cataldo and Nixon, 1990; Hook et al., 2020). Several studies have shown elevated levels of cathepsin B protein or its activity in plasma, cerebrospinal fluid, and amyloid plaques in individuals with AD. It is highlighted that increased plasma concentration of cathepsin B is associated with cognitive dysfunction in AD, as well as severe brain lesion outcomes (Sun et al., 2015).

In patients with AD, serum levels of cathepsin B were increased compared to healthy controls of the same age group. Importantly, the rise in cathepsin B has been correlated with dementia scores and cognitive impairment in the Mini-Mental State Examination (MMSE) in AD patients. Elevated plasma cathepsin B has been observed in both mild and severe AD compared to healthy controls. In patients with mild cognitive impairment (MCI), plasma levels of cathepsin B were similar to those of controls. These results indicate that increases in plasma cathepsin B are associated with the cognitive status of AD and occur in both early and advanced stages of the disease (Morena et al., 2017).

The results of these investigations support the hypothesis that the release of cathepsin B from lysosomes to the cytosol contributes to neurodegenerative processes and behavioral deficits in AD. These findings underscore the importance of exploring cathepsin B as a potential therapeutic target for the development of innovative treatment strategies targeting AD (Hook et al., 2020).

The study conducted by Mueller-Steiner where Cathepsin B was inhibited through genetic inactivation of the enzyme in mice expressing human familial Alzheimer’s disease mutant APP, resulted in increased levels of intracellular Aβ1-42, worsening plaque deposition and other Alzheimer’s disease-related pathologies. In contrast, when Cathepsin B was present, it reduced levels of Aβ42 peptides through proteolytic cleavage and decreased preexisting amyloid deposits. Furthermore, Cathepsin B cleaved both fibrillar and non-fibrillar assemblies of Aβ42 into shorter Aβ peptides (Mueller-Steiner et al., 2006).

It was also found that Cathepsin B is capable of cleaving the C-terminal portion of Aβ42, generating truncated Aβ peptides that are less amyloidogenic and more easily degraded by other proteases. Therefore, Cathepsin B likely plays anti-amyloidogenic and neuroprotective roles. Insufficient Cathepsin B activity may promote AD, whereas increasing Cathepsin B activity could potentially neutralize the neuropathology of this disease (Mueller-Steiner et al., 2006).

Cermak et al. (2016) confirmed these findings and demonstrated that cathepsin B regulates lysosomal function and intracellular cholesterol trafficking, which is crucial for the autophagy-lysosomal pathway. In *in vitro* experiments, enzyme inhibition with 100 μM PADK compromised this pathway, leading to alterations in LC3II, NPC1, and NPC2 proteins. Additionally, cathepsin B degraded Aβ as well as the C-terminal fragment of APP, facilitating subsequent degradation by BACE-1.

It is important to highlight that Cathepsin B contributes to the global degradation of Aβ in the brain, targeting different forms of Aβ due to its distinct proteolytic properties and subcellular localizations (Mueller-Steiner et al., 2006). These findings suggest that increasing endogenous Cathepsin B activity could potentially reduce Aβ levels, particularly Aβ1-42, and protect against Alzheimer’s disease-related deficits (Sun et al., 2008).

Genetic linkage studies have shown an association between a CST3 polymorphism and increased risk of late-onset sporadic Alzheimer’s disease (AD), supported by subsequent systematic meta-analyses (Crawford et al., 2000; Finckh et al., 2000; Beyer et al., 2001; Bertram et al., 2007). In the study by Sun et al. (2008), it was demonstrated that CysC is a key inhibitor of Cathepsin B-dependent Aβ degradation *in vivo*. The relative abundance of Aβ42 is modulated by the CysC-CatB axis, and Cathepsin B-induced truncation of Aβ42 is typically suppressed by endogenous CysC *in vivo* (Sun et al., 2008).

The authors observed that the significant reduction in plaque burden induced by CysC ablation depends on Cathepsin B. These intriguing effects could reflect the different mechanisms by which CysC regulates soluble and insoluble Aβ—whereby it inhibits Cathepsin B for the former and binds to Aβ for the latter (Kaeser et al., 2007; Mi et al., 2007). Therefore, in mice with reduced CysC levels, there is an increase in Cathepsin B activity, resulting in a marked decrease in overall Aβ deposits (Sun et al., 2008).

The increase in intracellular CysC may impair Cathepsin B-dependent degradation of Aβ in the endosomal/lysosomal pathways and contribute to Aβ accumulation. The finding that CysC regulates soluble Aβ by inhibiting Cathepsin B could lead to new, highly specific strategies for reducing Aβ (Sun et al., 2008).

In addition to its role in proteostasis, cathepsin B plays an important function in the brain’s immune system. Microglia express higher levels of cathepsin B compared to other cell types, and cathepsin B can promote the elimination of Aβ through microglia-mediated phagocytosis, cleaving internalized Aβ aggregates (Mueller-Steiner et al., 2006).

Besides, the cathepsin B activity is frequently elevated in inflammatory neurological diseases and it has relation with the alterations caused by inflammatory activity and it could result in organ failure (Hook et al., 2020).

The excessive concentration of Cathepsin B disrupts the microglial clearance functions, mainly in the hippocampal area, and these factors stimulate the IL-1β liberation, which intensifies the inflammation activity. Moreover, a cathepsin B inhibitor, CA-074, reduced the inflammatory response and neuronal death mediated by Aβ, which shows the importance of the enzyme in reducing neurodegeneration (Sun et al., 2008).

Table 1 summarizes the key alterations in proteostasis, autophagy, and lysosomes in AD, as discussed in this review.

**TABLE 1:** Summary of the key alterations in proteostasis, autophagy, and lysosomes in AD, as discussed in this review.

Concepts	Alterations in AD
Aβ formation	Generated extra or intracellularly from amyloid precursor protein (APP) by β or γ-secretases (Meng et al., 2024). Extracellular Aβ enters the cell through receptors (Zhang et al., 2023).
Aβ40 or 42 in the cells	Oligomeric peptide and plaque are recognized as abnormal, and in the cytosol, they undergo ubiquitination to be targeted either to the proteasome for degradation or to chaperone-mediated autophagy (CMA) (Choy et al., 2012; Mputhia et al., 2019).
Tau protein	Hyperphosphorylated Tau is delivered to lysosomes by chaperone-mediated autophagy (Kunz et al., 2004).
Proteostasis	Alterations in the ubiquitin-proteasome system, autophagy, and chaperones lead to the accumulation of protein aggregates and neuronal degeneration (Choy et al., 2012; Mputhia et al., 2019).
The ubiquitin-proteasome system activity	Aβ and Tau oligomers impair the proteasome activity, altering its localization and leading to the accumulation of polyubiquitinated substrates (Tai et al., 2012). Continuous proteasome inhibition results in excessive activation of the lysosomal system, increasing autophagy (Ihara et al., 2012).
Chaperone-mediated autophagy	Decreased levels of heat shock protein Hsp70 contribute to these alterations (Goetzl et al., 2015).
Autophagy	Multiple levels within the endolysosomal system are affected in AD patients (Goetzl et al., 2015; Tramutola et al., 2015). Intracellular Aβ and misfolded Tau protein are internalized into autophagic vacuoles containing ApoE and chaperone-mediated autophagy, respectively, for subsequent degradation (Fuentealba et al., 2010; Caballero et al., 2018). Increased levels of LAMP-1, alterations in mTOR signaling and decreased expression of the autophagy regulator Beclin1 reduce autophagy (Goetzl et al., 2015; Tramutola et al., 2015). Endosomes enlarged, with Rab5 within them, instead of the cytosol, alongside Rab4 (Tramutola et al., 2015).
Lysosomes	Lysosomal rupture, associated with the genetic risk factor related to ApoE4, destabilizes lysosomal membranes, leading to lysosomal leakage and apoptosis (Perez et al., 2014).
Lysosomal enzymes	Dysfunction of the v-ATPase causes alkalinization of the organelle, compromising the catalytic activity of intralysosomal enzymes (Mindell, 2012).
Cathepsin D	Abnormal distribution and increased levels of the enzyme are found in extra-lysosomal localizations in the brain (Nakamura et al., 1991). It can process APP in specific residues, with an increased velocity of the reaction in mutated APP (Vidoni et al., 2016; Higaki et al., 1996; Dreyer et al., 1994; Gallwitz et al., 2022). It can degrade oligomeric Aβ42, which is subsequently secreted by exocytosis (Gallwitz et al., 2022).
Cathepsin B	Levels increased and abnormal distribution in extra lysosomal compartments (Nakamura et al., 1991). It can degrade oligomeric Aβ42 and is subsequently secreted by exocytosis (Mueller-Steiner et al., 2006). It is associated with the cognitive status of AD and occurs in both early and advanced stages of the disease (Morena et al., 2017). Regulates lysosomal function and intracellular cholesterol trafficking (Cermak et al., 2016).

## 7 Novel drugs for autophagy modulation in Alzheimer’s

Few clinical studies are currently underway to restore the autophagy-lysosomal system. One such study involves hydralazine, which is in phase 3 clinical trials for mild-to-moderate AD and, among various mechanisms, activates autophagy (Huang et al., 2023). Nilotinib, an FDA-approved drug for chronic myeloid leukemia, is in phase III clinical trials for AD due to its effect on increasing autophagy. Animal studies have shown that this increase in autophagy led to a decrease in amyloid plaques and an increase in cognition (Nobili et al., 2023).

In phase II clinical trials, GSK4527226, a monoclonal antibody against the sortilin protein (SORT1), increased lysosomal function. Rapamycin, also in phase II clinical trials, increases autophagy and inhibits mTOR (NCT04629495).

However, many compounds show, in clinical studies, the disaggregation of amyloid plaques, fibrils, or oligomers, leading to improved memory and cognition, but with mechanisms not yet fully elucidated. Restoration of this system, along with de-oligomerization, may be the mechanism underlying this improvement (Cummings et al., 2024).

While monoclonal antibodies have shown efficacy in reducing amyloid burden in Alzheimer’s disease, the underlying mechanisms of action may involve the autophagy-lysosomal system. Pre-clinical and clinical studies support the notion that compounds targeting this system can enhance clearance of toxic protein aggregates, suggesting a potential synergistic effect when combined with amyloid-directed therapies.

## 8 Conclusion

Lysosomal dysfunction, characterized by impaired clearance and biogenesis, plays a pivotal role in the pathogenesis of AD by exacerbating protein aggregation, characteristic of the disease. The intricate interplay between autophagy and the endosome-lysosomal system is essential for maintaining cellular homeostasis and is compromised in AD. Strategies that enhance lysosomal clearance or enhance lysosomal biogenesis are thus emerging as potential therapies (Nixon, 2013).

It is well established that the increased levels of Aβ and protein-Tau can impair autophagic and mitochondrial function, contributing to disease pathogenesis (Xu et al., 2024). While there is compelling evidence supporting the involvement of the lysosomal pathway in AD, the precise mechanisms, particularly the role of cathepsins, remain to be fully elucidated. Further research is necessary to clarify the contributions of lysosomal enzymes to disease progression and to identify novel therapeutic targets. Enhancing lysosomal function and promoting proteostase represent promising avenues for the development of effective treatments for AD.
